# Biofilm and Small Colony Variants—An Update on *Staphylococcus aureus* Strategies toward Drug Resistance

**DOI:** 10.3390/ijms23031241

**Published:** 2022-01-22

**Authors:** Henan Guo, Yucui Tong, Junhao Cheng, Zaheer Abbas, Zhongxuan Li, Junyong Wang, Yichen Zhou, Dayong Si, Rijun Zhang

**Affiliations:** Laboratory of Feed Biotechnology, State Key Laboratory of Animal Nutrition, College of Animal Science and Technology, China Agricultural University, Beijing 100193, China; ghn_657@cau.edu.cn (H.G.); 15956910334@163.com (Y.T.); chengjunhao@cau.edu.cn (J.C.); Zaheerabbas113@yahoo.com (Z.A.); Lee_zx20@yeah.net (Z.L.); wangjy9722@163.com (J.W.); redhoh@163.com (Y.Z.); dayong@cau.edu.cn (D.S.)

**Keywords:** drug-resistance, multidrug-resistance, *Staphylococcus aureus*, biofilm, small colony variants, global regulation, resistant mechanisms

## Abstract

Recently, the drawbacks arising from the overuse of antibiotics have drawn growing public attention. Among them, drug-resistance (DR) and even multidrug-resistance (MDR) pose significant challenges in clinical practice. As a representative of a DR or MDR pathogen, *Staphylococcus aureus* can cause diversity of infections related to different organs, and can survive or adapt to the diverse hostile environments by switching into other phenotypes, including biofilm and small colony variants (SCVs), with altered physiologic or metabolic characteristics. In this review, we briefly describe the development of the DR/MDR as well as the classical mechanisms (accumulation of the resistant genes). Moreover, we use multidimensional scaling analysis to evaluate the MDR relevant hotspots in the recent published reports. Furthermore, we mainly focus on the possible non-classical resistance mechanisms triggered by the two important alternative phenotypes of the *S. aureus*, biofilm and SCVs, which are fundamentally caused by the different global regulation of the *S. aureus* population, such as the main quorum-sensing (QS) and agr system and its coordinated regulated factors, such as the SarA family proteins and the alternative sigma factor σB (SigB). Both the biofilm and the SCVs are able to escape from the host immune response, and resist the therapeutic effects of antibiotics through the physical or the biological barriers, and become less sensitive to some antibiotics by the dormant state with the limited metabolisms.

## 1. Introduction

In the past decades, antibiotics have played a significant role in reducing the risks involved in childbirth, injuries, and intrusive medical procedures [1,2]. However, abuse or overuse of antibiotics in the circumstances of experimental studies and clinical treatments poses a severe threat to public health by acquiring drug-resistance (DR) and multidrug-resistance (MDR) of the pathogens [3,4]. It is of great concern that if the growing resistance to antibiotics continues, the global economies will suffer a sharp loss of USD 100 trillion by 2050 [5].

Among the DR/MDR pathogens, *Staphylococcus aureus* is an all-powerful pathogen that can cause acute, persistent, and chronic infections [6]. The failure of antibiotics in the clinical treatment is a complex outcome of the accumulation of resistance-relevant genes and the internal tolerant nature of the flexible phenotype changes of *S. aureus,* including biofilm formation and small colony variants (SCVs) [7]. Phenotypic change is a part of the normal growth cycle of the bacteria [8], and acts as an insurance approach to avoid harmful environments [9]. With the help of these alternative phenotypes, *S. aureus* can survive and adapt to the corresponding environments, and further invade the host body [7]. It is known that the development of these novel phenotypes has been involved in chronicity and relapse in *S. aureus*-relevant infections [10], such as chronic osteomyelitis [11,12], chronic rhinosinusitis [13,14], soft tissue infections [15,16], endocarditis [17,18], sepsis [19,20], and medical device-related infections [21]. Infections caused by either biofilm or SCVs have certain common features, including the failure of host defenses arising from their dormant state, and the inherent tolerance of antibiotics [7,22]. Behaviors of *S. aureus* population are generally controlled by a global regulation system, such as the quorum-sensing (QS) system, which is concerned as a population density-dependent and environment-dependent regulatory pathway to ensure intercellular communication [23]. Therefore, some special regulations allow alternative lifestyles, such as biofilm and SCVs, to form the individual physiology, metabolic, and pathogenic characteristics to adapt to the altered living environments. In turn, those special features enable biofilm and SCVs to combat the host immune effect and even resist the therapeutic effect of the common antibiotics.

In this review, we focus on the development of DR/MDR and the classical resistant mechanisms associated with the accumulation of the resistance genes and analyze the hotspots of the recently published reports with keywords “MDR/multidrug-resistance/multidrug-resistant” by multidimensional scaling analysis. Furthermore, this review seeks to give a comprehensive overview of the formation, the critical global regulatory pathways, and the possible drug-resistance mechanisms of the *S. aureus* alternative phenotypes, biofilm and SCVs.

## 2. The Development of DR or MDR

With the discovery of antibiotics, humans once got rid of some infections caused by pathogens. However, during the past decades, the armed pathogen has rendered lots of antibiotics insensitive and ineffective, even vancomycin, the last defense against multi-resistant bacteria, cannot avoid the same fate [24]. During the pathogens’ endless coevolutionary process with humans, antibiotics arguably become the most powerful selective pressure on drug-resistance [25]. For instance, the history of *S. aureus* drug resistance is listed in Table 1. As shown, the rise of new antibiotics could figure out the current epidemics around the world caused by *S. aureus*. However, it is also an inevitable trend that the drug resistance spectrum of *S. aureus* can be widened.

**Figure 1:**
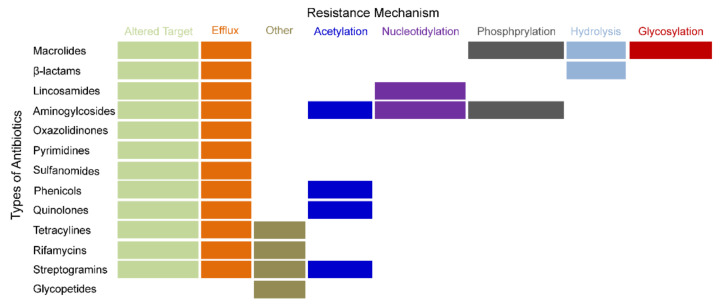
Classical drug-resistant mechanisms of common antibiotics. This figure was modified based on the Figure 1 of Dunn, et al. [31]. Labels in different colors stand for various drug-resistant mechanisms.

Pathogens can develop resistance to most effective antibiotics in clinical treatment, and further, lead to urgent issues to the global human health [31]. It is accepted that the mechanisms of drug resistance are diverse and complicated. The majority of the classical drug resistance mechanisms are shown in Figure 1 [31]. These resistance mechanisms arise from pathogen chromogene mutations and the acquisition of resistance genes from other strains during the horizontal gene transfer (HGT) process [32].

Apart from the defects in the clinical treatment, it also has been reported that antibiotics used in veterinary practices are positively associated with resistance genes in both pigs and poultry [33]. In addition, the microbes of livestock have been proven to disturb human health by delivery of resistant bacteria and genes HGT [34]. Last but not least, the overuse of antibiotics can also result in environmental pollution, reflected as the ubiquitous detectable rate of antibiotics in natural ecosystems, especially fresh waters, which might aggravate drug resistance and destroy the food chain by toxicity in the waters [35].

## 3. The Hotspots of MDR Relevant Recent Studies

The ever-increasing population of MDR pathogens has become a tough challenge menacing global public health care systems [36]. MDR is known as microbial resistance against at least two antimicrobial agents that used to be sanitary [37]. Antibiotic-resistant infections are capable of inducing morbidity and raising mortality [38,39,40]. The majority of nosocomial infections are caused by the ESKAPE pathogens, including *Enterococcus faecium*, *S. aureus*, *Klebsiella pneumoniae*, *Acinetobacter baumannii*, *Pseudomonas aeruginosa*, and *Enterobacter* species, most of which possess multidrug-resistance [3]. In particular, the rise of super-bacteria has been observed for a while, which sounds another alarm for global health [41].

**Figure 2:**
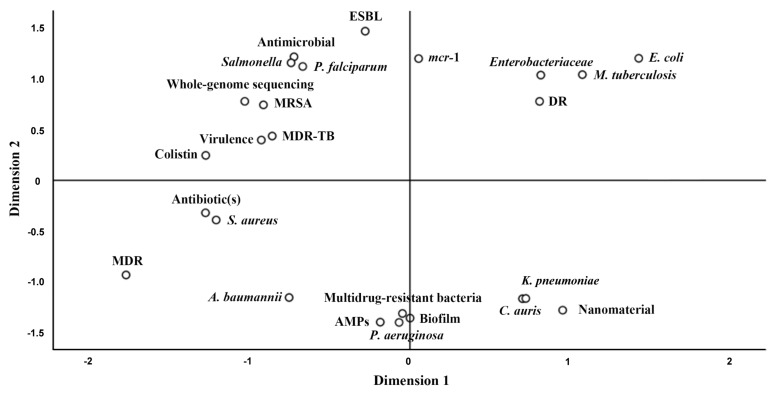
Multidimensional scaling (MDS) of MDR-related reports in the Web of Science core collection.

Recently, studies on MDR have been mounting, containing several aspects. From January to June 2021, 2666 articles with the keywords “MDR/multidrug-resistance/multidrug-resistant” (excluding cancer-related reports) have been included in the Web of Science core collection. SATI software was used to analyze and evaluate hotspots, frequencies, and relationships of the targeted research [42]. Based on the results of SATI, multidimensional scaling analysis can discover the topic distribution of the target studies by measuring the distance between different keywords [42]. The distances of dots indicate the relationship of each keyword, namely, the closer the distance, the stronger the correlation. Figure 2 demonstrates the results of the multidimensional scale analysis of the top 25 keywords in the MDR-related studies. The keywords could be classified into several classes: DR/MDR pathogens (*P. aeruginosa*, *Enterobacteriaceae*, *E. coli*, *A. baumannii*, *Salmonella*, *S. aureus*, MRSA, *M. tuberculosis*, *Klebsiella pneumoniae*, *C. auris*, MDR-TB, and *P. falciparum*); resistant antibiotic or factors [colistin, extended-spectrum β-lactamases (ESBL), and *mcr-1*]; MDR pathogen-related characteristics (virulence and biofilm); new antibiotic alternatives (AMPs and nanomaterial); and a useful drug-resistance gene detection method whole-genome sequencing. We can conclude that, for the past 6 months, over 2000 articles related to different DR/MDR pathogens have been reported, proving again that MDR has become a crucial problem that needs to be handled. As shown in Figure 2, *S. aureus* was the closest one from MDR among all reported MDR pathogens, which suggested that many studies have concentrated on the connection between *S. aureus* and MDR. In this review, we will focus on the unconventional possible resistance mechanisms of *S. aureus*.

## 4. Alternative Typical Phenotypic Switches of *S. aureus*

*S. aureus* is an all-powerful pathogen connected to various clinical syndromes, ranging from acute sepsis to more persistent chronic infections related to endocardium, lung, marrow, skin, and soft tissues [6]. These infections can be difficult to cure due to the increased tolerance of *S. aureus* to antibiotics [43]. Consequently, *S. aureus* is also concerning as a human pathogen responsible for high morbidity and mortality worldwide [6,44].

*S. aureus* has been proven to to be invading and surviving in both professional and non-professional phagocytes [45,46]. Then, *S. aureus* may undergo three distinct fates: *S. aureus* escapes from the phagosome, proliferates inside the host cells that tend to be finally killed by the bacteria; *S. aureus* is killed by the host defense reactions; and it remains alive intracellularly without being eliminated [45].

Intracellular localization can protect *S. aureus* from the host immune system and treatment of antibiotics [47]. Unlike the classical resistance mechanisms by the acquisition of new virulence genes, the phenotypes existing within a genetically clonal population confer tolerance to antibiotics, which are defined as alternative lifestyles with limited or no growth, but are survival cells [43,48,49]. Although the mechanisms might be distinct, these alternative lifestyles tend to form dormant subpopulations to improve adaptation in unfavorable conditions [43], which are difficult to clear up by the host immune system or other therapeutic methods, and can revert to their original parental and active type at the right time [50,51]. It is known that small colony variants (SCVs) and biofilm are two typical phenotypic switches of *S. aureus* [43].

### 4.1. An Introduction of Biofilm Phenotype

#### 4.1.1. Formation of *S. aureus* Biofilm

It is estimated that over 60% of treated bacterial infections arise from biofilm in developed countries [52]. Implantable medical devices have become an important part of modern healthcare, but they are also facing the challenge of biofilm-relevant infections caused by surface-adhering bacteria, especially *S. aureus* [53].

Biofilm is a biotic or abiotic surface-dependent multicellular lifestyle that embeds bacteria in its extracellular matrix composed of extracellular polymeric substance (EPS), proteins, and extracellular DNA [54]. Usually, the formation of biofilm consists of the following stages: attachment of planktonic cells to a substratum surface; colonization, fertilization, and biofilm mature; biofilm maturation; and biofilm dispersal [55], as shown in [54]. As soon as the biofilm grows into a mature three-dimensional structure, the community of the biofilm disassembles and releases the planktonic cells, which are ready to repeat the process to form a new biofilm [54]. Generally, the growing cells within biofilms are classically divided into four metabolic states: aerobic (located in the outer layers with sufficient oxygen and nutrients), fermentative (located in the inner layers with lack of oxygen and nutrients), dormant (located in anoxic layer with slow growth and inactive metabolism) and dead, among which the dormant cells dominate and have potential to eventually become persister cells [56]. *S. aureus* biofilm protects the cells from hostile conditions, including extreme temperature, limitations of nutrients and dehydration, and even antibacterial drugs [52,57].

The formation and the bioactivities of biofilm are determined by the fundamental components. In terms of the composition of biofilm, the majority of EPS components within *S. aureus* biofilm is the polysaccharide intercellular adhesin (PIA) [58], which plays an important role in several aspects, including colonization, biofilm generation and biofilm-associated infections, immune evasion, and resistance to antimicrobials and phagocytosis [59]. What is more, *S. aureus* EPS also connects to the following proteins with different effects: accumulation-associated proteins (Aap) interact with PIA to facilitate the maturation of biofilm [58]; the surface-binding proteins, Spa and SasG, are related to surface attachment and infections [60]; the fibronectin-binding proteins, FnbA and FnbB, can afford the pathogen the ability to attach to cellular integrins and further trigger the internalization into the host cells [61]; and the cell wall-anchored (CWA) proteins facilitate the adhesion among EPS, the membrane of the host cell, and the CWA proteins on adjacent cells, thus promoting the accumulation of biofilm [62]. Furthermore, it is accepted that amyloid fibers can provide a scaffold for biofilm to form a basis of matrix and maintain its structural stability [63,64]. Except for the above components, eDNA has also been proven to be crucial in the formation of biofilm by facilitating the irreversible attachment, inducing the HGT progress, maintaining the integrity of biofilm, increasing the tolerance of drugs, and guaranteeing the evasion from the host’s innate immune system [65].

#### 4.1.2. Global Regulation of *S. aureus* Biofilm Formation

The development of biofilm is a population behavior that is tightly controlled by a complex global regulatory system. One of the main regulatory systems is the quorum-sensing (QS) system [66]. The QS system is responsible for intercellular communication of bacteria by modulating the population behavior in a density-dependent manner [67]. The agr system, the most essential part of the QS system of *S. aureus*, regulates the expression of about 150 genes encoding adhesins and virulence factors as well as the accumulation of extracellular auto-inducing peptides (AIPs) to control the cell population density [68]. The multifunctional agr system consists of two different operons, RNAII and RNAIII, activated by the promoters P2 and P3, respectively [69].

The operon RNAII contains agr BDCA genes, encoding AgrBDCA proteins, respectively [70]. AgrD, the precursor of AIP, is produced and exported on the plasma membrane with the aid of AgrB and Spsb (signal peptidase IB) [70]. Once AIP reaches the threshold concentration, it will bind to the receptor of the membrane-bound histidine kinase AgrC, followed by the activation of the AgrC kinase [71]. Finally, the response regulator AgrA is phosphorylated by AgrC to initiate the signaling cascade process, which in turn produces a positive feedback loop on P2 and P3 promoters [70]. RNAIII, an important effector of the agr system, can upregulate genes encoding exoproteins including toxins, haemolysins and exoproteases; meanwhile, it is also responsible for the downregulation of several genes encoding surface-related adhesins, such as fibronectin-binding proteins and serine-aspartate repeat family proteins [71,72] Additionally, the phenol-soluble modulin (PSMs) family and matrix-degrading enzymes in *S. aureus* are directly upregulated by AgrA [71,73,74], which are known to facilitate the maturation and disassembly of biofilm [75].

**Figure 3:**
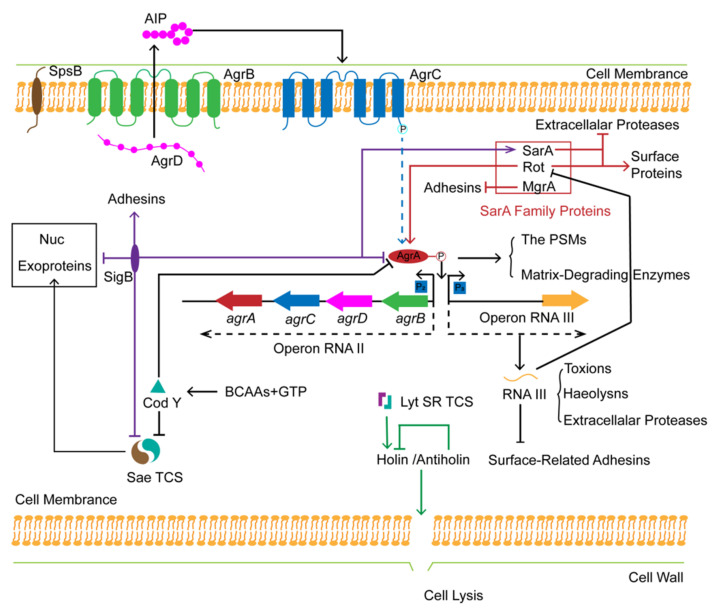
Global regulation of *S. aureus* biofilm formation. This figure was modified based on Figure 2 of Schilcher et al. [54].

Except for the agr system, there exist several important regulatory factors to participate in the formation of biofilm, which can interact or cooperate with the agr system in the global regulation of *S. aureus*, as shown in Figure 3 (modified based on Figure 2 of Schilcher et al. [54]).

- The Sae and the LytSR Two-Component Systems

Among complex regulators of the *S. aureus* global regulation system, there exist several two-component systems, such as the *S. aureus* exprotein expression (Sae) two-component system (TCS) and LytSR TCS. Namely, the complete function of the system is controlled by two different but related elements.

The *S. aureus* exprotein expression (Sae) two-component system (TCS) contains a sensor histidine kinase SaeS and a response regulator SaeR [76]. The former component controls the expression of several exoproteins during biofilm formation, such as a-hemolysin (Hla), FnbA, FnbB, and the extracellular thermonuclease Nuc (cleaving the eDNA contained in the matrix) [77,78,79,80,81].

Cell death and lysis of *S. aureus* are controlled by the holin/antiholin system [82] encoded by cidA and IraA, respectively [83,84]. The holin can improve extracellular murein hydrolase activity resulting in cell lysis, which is antagonized by antiholin [84,85]. The biosynthesis of holin/antiholin is tightly controlled by the LytSR TCS [86].

- SarA Family Proteins

The formation of biofilm is also controlled by the Staphylococcal accessory regulator (SarA) family proteins. Herein, we briefly describe three members of the SarA family, SarA, Rot, and MgrA. SarA, a general transcriptional factor, can directly upregulate the expression of exoproteins [87] and the agr system [88], whereas it can downregulate the extracellular proteases during the biofilm formation [89]. Rot can induce the expression of surface proteins and repress the production of extracellular enzymes [90,91,92], which is suppressed by the effector RNAIII of the agr system [93,94]. MgrA acts as a negative regulator of biofilm formation by repressing the production of adhesins [95].

- Alternative Sigma Factor σ^B^ (SigB)

Biofilm formation is a natural survival strategy for *S. aureus* to adapt to hostile environments, which means that the pathogen population needs to respond to the stress quickly. Due to the regulation of stress-relevant genes by the induction of the transcriptional alternative sigma factor σ^B^ (SigB), it is possible for *S. aureus* to survive in extreme conditions [96]. Moreover, SigB can promote the initial stage of biofilm formation by increasing the biosynthesis of adhesins and can repress the dispersal of the mature biofilm by decreasing the transcriptional level of the thermonuclease Nuc and proteases [97,98,99,100,101]. What is more, SigB can indirectly regulate the biofilm formation through influencing the other regulatory systems, for instance, by inhibiting the activity of RNAIII and SaeRS TCS, and upregulating the SarA expression based on the corresponding environments [102,103,104].

- The Transcriptional Repressor CodY

Usually, biofilm formation is controlled by the nutritional availability and the metabolic state of the bacteria [54]. *S. aureus* can fit in the stationary growth phase and starvation by modulating over 100 genes expression related to primary metabolism, transport, and virulence via a DNA-binding factor, CodY, which is triggered by a nutritional sufficient threshold of branched-chain amino acids (BCAAs) and GTP [105,106,107]. Besides, depressed CodY can induce the increased level of thermonuclease Nuc by the Sae TCS, resulting in the cascading regulation of toxins and the generation of factors [108]. What is more, CodY is also essential in the early stage of biofilm formation by strongly repressing the agr system [109].

#### 4.1.3. Drug-Resistance Mechanisms Induced by Biofilm of *S. aureus*

Under global regulation, the biofilm of *S. aureus* forms, which empowers the microbial population to increase the tolerance of the antibiotics. As biofilm effectively shields bacteria from harsh host environments, it is complicated to explain the drug resistance mechanisms of biofilm [53]. Previous studies revealed several reasons for the drug resistance of biofilm, including its physical barrier, physiology states, oxygen availability, and genome adaptability [110,111,112]. One of the resistance mechanisms is related to the structural characteristics of the biofilm matrix itself. During the process of infection, the biofilms provide a strong safeguard from handicaps, including the host immune response and antibiotic therapy. As biofilm acts as a strong physical barrier to conceal pathogen-associated molecular patterns (PAMPs), those pathogens are capable of resisting the host immune response for a long time [44,62,113,114]. Moreover, biofilm can also protect the invading bacteria from the immune system of the host by disrupting the activation of phagocytes and the complement system [115,116]. Similarly, due to the limited diffusion and repulsion of antibiotics under such a complicated matrix, it is hard for drugs to get access to the target, thus allowing the generation of drug resistance [117,118,119]. It has been reported that, compared to the planktonic cells, bacteria within the biofilm can increase the tolerance against the conventional antibiotics by about 1,000-fold [58,120,121]. Furthermore, the physiological state of the bacteria within biofilm also contributes to the altered tolerance of antibiotics. Due to the lack of nutrients or oxygen, bacteria within the biofilm, particularly those deep within the matrix, tend to turn into a dormant state or persister cells, of which the limited growth can reduce the efficacy of antibiotics targeting the active cell processes [122,123,124]. Dormant cells grow slowly with a limited metabolism state, resulting in reduced intracellular ATP concentration, which makes bacteria less sensitive to antibiotics [125,126,127]. What is more, suffering from several times of treatment by antibiotics, cells in dormancy state remain alive and ready to continue the formation of biofilm [122]. Last but not least, cells in biofilm undergo a higher rate of resistance-relevant mutation than planktonic cells [128].

### 4.2. An Introduction of Biofilm Phenotype

#### 4.2.1. Formation of SCVs

**Figure 4:**
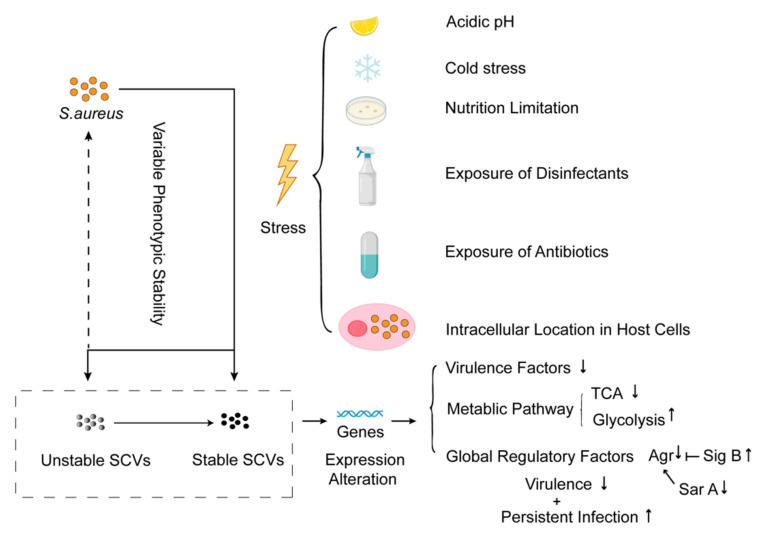
Formation and characteristics of *S. aureus* SCVs.

*S. aureus* SCVs first attracted public attention due to the association with chronic and recurrent infections in 1995 [129]. Recently, SCVs have been proven to be an essential cause for infections in different organs [130,131,132]. It was reported that *S. aureus* SCVs were found in 29% of osteomyelitis clinical cases [133] and 17-46% of chronic cystic fibrosis cases [134,135,136]. In particular, prolonged survival of *S. aureus* within the host cells has been associated with an increasing percentage of SCV sub-population [7]. SCVs, with the small colony and variable phenotypic stability, have been defined as a type of slow-growing bacterial sub-population formed under a certain selective pressure, including extreme pH, cold stress, nutrition limitation, exposure to antibiotics or disinfectants, and location or survival in the host cells [137,138,139] (Figure 4). *S. aureus* SCVs are always characterized by reduced pigment and haemolysis [22,140]. Moreover, most of the SCVs display other features, containing the repressed tricarboxylic acid cycle (TCA) cycle, the improved glycolysis, and the depressed global regulator factor AgrA, which eventually leads to reduced virulence and strengthens the persistent infections of SCVs [138,139].

#### 4.2.2. Global Regulation of SCVs Formation

Unlike the global regulation of *S. aureus* biofilm, the counterpart of SCVs demonstrates a trend with a repressed agr system. In detail, the depressed agr system in SCVs is a combination of increased negative regulators (e.g., SigB), decreased positive regulators (e.g., SarA), or the degraded function of RNA degrasome on the AgrA effector RNAIII [138,141,142,143]. As many of toxins are controlled by RNAIII, its downregulation leads to the persistent infection of SCVs without elimination by the host immune systems [144]. It is accepted that the persistent infections of *S. aureus* are strongly associated with the decrease or loss of essential virulence factors regulated by the agr system and RNAIII [138]. Meanwhile, the main features of SCVs, such as the increased cell-surface proteins and the repressed exotoxins, are related to the increased level of SigB [12], which also contributes to the persistent infections. It was demonstrated that *S. aureus* with a mutation in SigB lost the ability to transform into the SCV phenotype and could be removed by the host immune response [100,145]. What is more, SigB can regulate another global regulator, SarA, which increases the expression level of hemolysins and FnbA/FnbB in an agr-dependent/independent way [146,147].

#### 4.2.3. Drug-Resistance Mechanisms Induced by SCVs

Similar to the effects of biofilm, during the cause of infection, the SCVs also provide a safeguard from handicap, including the host immune system and antibiotic therapy. During infection, pathogens can usually stimulate the host immune response by reorganization between bacterial-specific antigens and PAMPs of the host cells [148]. However, compared to the wild *S. aureus*, SCVs with the dormant state tend to induce more attenuated host immune response during the infection of host cells, reflected by decreased chemokine and adhesion molecules [22,144], and eliminate effects of antimicrobial peptides inside the host’s innate immune system [149,150]. Consequently, escaping from the severe host response, SCVs can function as a reservoir for high invasion and persistent infection inside the host cells [139]. What is more, it is possible for some intracellular-located SCVs to revert to the parental wild-type phenotype in a nutritionally rich environment or without selective pressure, called unstable SCVs, thus resulting in a dynamic *S. aureus* population [8,151].

**Figure 5:**
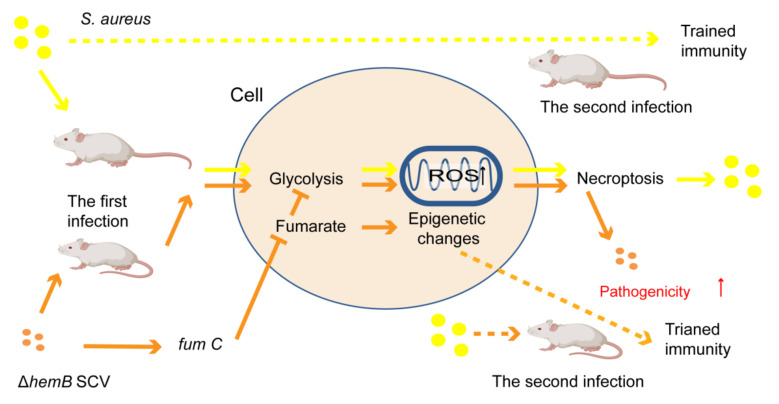
Effects of *S. aureus* ΔhemB SCV infection to mice. This figure was drawn according to the report of Lung et al. [153].

Moreover, SCVs can also lead to the disabled defense of the hosts’ innate immune systems by altering the host metabolism, such as glycolysis, which is related to some critical immunologic functions [152]. In detail, SCVs often contain inactivating mutations in genes associated with the generation of the terminal electron transport chain components, haeme and menaquinone [153]. It was reported that both wild-type *S. aureus* and ΔhemB SCV (carrying a mutation in the hemin biosynthetic pathway) could promote glycolysis in host cells with the accumulation of mitochondrial reactive oxygen species (ROS) to induce caspase-independent host cell necroptosis without elimination of the microbial cells [153] (Figure 5). However, to maintain glycolysis, the ΔhemB SCV induced the expression of gene fumC encoding an enzyme to degrade a host glycolysis inhibitor fumarate, resulting in the inactivation of the epigenetic changes connected with trained immunity during secondary *S. aureus* infection of the host [153,154], with effective increase pathogenicity of ΔhemB SCV [153].

Due to the fact that SCVs possess those special features, including depressed metabolism, slow proliferation, and drug-resistance, there exist limited therapies to clinically combat SCVs [139]. Several studies have proved that *S. aureus* SCVs display an elevated antibiotic tolerance, which is independent of the accumulation of resistance genes [8,155]. Infections arising from dormant sub-population of bacteria with special metabolism and growth states will induce phenotypic tolerance of antibiotics. As an example, there are menadione- and haemin-auxotroph SCVs, which are typical types of SCVs induced by aminoglycosides antibiotics [7]. Generally, menadione and haemin are essential for the biosynthesis of cytochromes and menaquinone in the electron transport system, which contributes to the production of ATP [156]. The generation of ATP is demanded for forming normal colonies by facilitating cell wall biosynthesis, pigmentation, and membrane potential, which can promote the uptake of antibiotics, such as aminoglycosides [157,158]. Therefore, decreased ATP in the menadione- and haemin-auxotroph SCVs leads to the increased resistance of cell wall-specific drugs and limited uptake of antibiotics that need large membrane electrochemical gradients [159]. In addition, bacteria with decreased ATP will contribute to growth dormancy and less active antibiotic targets, followed by less damage under exposure to antibiotics, which accounts for the increasing drug tolerance [7]. Finally, host cells provide a shelter for SCVs to block the uptake of drugs with limited ability to cross the eukaryotic membranes [160].

## 5. Conclusions and Future Recommendations

Compared to most of the other reviews revealing the classical acquired drug resistance caused by the accumulation of the resistant genes, this review provides some new insights of *S. aureus* drug resistance from aspects of alternative phenotypes, including biofilm and SCVs, innovatively. Herein, we focus on the natural potential of *S. aureus* drug resistance.

Overall, accompanied by the usage of antibiotics, the development of the DR/MDR continues. However, it is complicated to understand the resistance of pathogens. To date, many studies have revealed the mechanisms of drug resistance. To take *S. aureus* as a resistant pathogen example, the acquired DR/MDR results from the accumulation of resistant genes or the altered physical or metabolic states of the special alternative phenotypes, biofilm and SCVs. The awareness of the public of those phenotypes is growing, along with increased studies about biofilm and SCVs in different fields. Despite the distinct forming reasons of SCVs and biofilm, the two lifestyles share similarities in certain features, such as the limited metabolism and growth, partial global regulation changes, and gene expression levels related to the extracellular surface components and virulence factors.

Both biofilm and SCVs are characterized by persistent infections, and growing resistance to antibiotics and antimicrobial agents, causing a severe burden on human health care. Consequently, the treatment of infections related to *S. aureus* alternative phenotypes is still a complicated challenge. With the development of biofilm research, there are many anti-biofilm agents that can inhibit the cycle of biofilm by penetrating the cell membrane of living pathogens, or block the development of biofilm through targeted disturbing of certain actions of QS systems. All those anti-biofilm agents can be classified as traditional natural active agents [161] and synthetic analogue anti-biofilm agents based on the biological and computational technology [162]. Furthermore, the increasing development of nano-materials also contributes to the abundance of the anti-biofilm agents that have shown great potential as promising bactericidal agents by physically destroying the bacteria cell membranes and further restraining the development of drug-resistant bacteria [163]. However, the development of anti-SCVs agents is comparatively underdeveloped. Until now, there have been few effective anti-SCVs agents found.

From the clinical perspective, we should pay more attention to chronic or persistent infections caused by biofilm or SCVs, due to their relapsing nature. Moreover, numerous studies are still in need of exploration to deal with the antibiotic resistance of *S. aureus* from the angle of the resistant phenotype-relevant regulatory pathways in certain infections. In short, this review provides a summary of the relationship between the alternative phenotypes and their native resistance mechanisms of antibiotics, which may help further investigations to combat the resistant pathogen in infections caused by the DR and MDR pathogen.

## Figures and Tables

**Table 1:** Different drug-resistance stages of *S. aureus*.

Resistant Stage	Occurrence Time	Mechanism	Drug Resistance	Typical Strain	Reference
Penicillin-resistant strains	In the mid- 1940s	Plasmid-encoded penicillinase hydrolysing the β-lactam ring of penicillin	Penicillin	Phage type 80/81 *S. aureus*	[26,27]
Methicillin-resistant *S. aureus* (MRSA)	1961	Gene *mecA* and *mecC* encoding the low-affinity penicillin-binding protein PBP2A or PBP2A_LGA_	Entire β-lactam class of antibiotics	MRSA COMRSAL	[28,29]
Vancomycin-resistant *S. aureus* (VRSA)	At the end of the 20th century	Mediated by *vanA* gene cluster	Vancomycin	N/A	[24,30]

## Data Availability

Not applicable.
